# Sex disparities in the risk of intracranial aneurysm rupture: a case–control study

**DOI:** 10.3389/fneur.2024.1483679

**Published:** 2024-12-27

**Authors:** Dong Shen, Miaochun Cai, Yi Luo, Zhihao Li, Peidong Zhang, Yongkang Wang, Wenlong Fan, Hanqiu Wu, Yezhou Yu, Xijun Gong, Chen Mao

**Affiliations:** ^1^Department of Epidemiology, School of Public Health, Southern Medical University, Guangzhou, China; ^2^Department of Diagnostic Imaging, Anhui Provincial Hospital, Hefei, China; ^3^Department of Radiology, The Second Affiliated Hospital of Anhui Medical University, Hefei, China

**Keywords:** sex difference, female, intracranial aneurysm, rupture, risk

## Abstract

**Background:**

There are sex disparities in the risk of ruptured intracranial aneurysm (IA), but which sex-specific factors are related to ruptured IA remains inconclusive.

**Methods:**

Data from electronic medical records from two tertiary hospitals, collected between January 2012 and December 2019, were analyzed for this study. All IAs were confirmed by computed tomographic angiography or digital subtraction angiography. Sex-specific factors associated with ruptured IA were analyzed using multivariable logistic models with a case–control study design. Age, aneurysm size, and aneurysm location subgroup analyses were conducted according to sex.

**Results:**

In total, 1883 patients [1,117 (59.32%) female, 766 (40.68%) male] with 2,423 IAs were included; 734 (38.98%) of patients had ruptured IAs. Compared with males, females had a higher risk of ruptured IA [odds ratio, 1.72 (95% confidence interval, 1.38–2.14)]. Age, aneurysm location, aneurysm size, multiple aneurysms, hypertension, history of intracerebral hemorrhage, and ischemic stroke were associated with risk of IA rupture in both sexes. In the subgroups based on the covariates used in this study, we only identified statistically significant interaction between sex and age. Although ruptured IAs were most common in males and females aged 50–59 and 60–69 years, respectively, risk of IA rupture peaked at ages 30 and 30–50 years in females and males, respectively, and decreased with age in both sexes.

**Conclusion:**

Females have an overall greater IA incidence and higher risk of IA rupture than males. Young age is one sex-specific risk factor associated with ruptured IA which could related to potential influence of hypertension, which might suggest more attention of IA rupture prevention in younger female.

## 1 Introduction

Intracranial aneurysm (IA) is a common cerebrovascular disorder, that represents an increasing disease burden worldwide (1). A systematic review and meta-analysis, including 68 studies and 94,912 patients from 21 countries, reported that the overall prevalence of unruptured IA was 3.2% [95% confidence interval (CI), 1.9–5.2%] in a normal population without comorbidities (2). Ruptured IAs are responsible for approximately 85% of cases of subarachnoid hemorrhage (SAH), which is associated with 30-day fatality rates reaching 36–42% and permanent disability rates of up to 50% (3, 4).

Women have a greater risk of IA incidence and rupture than men (5–8), with a reported female and male incidence ratio of 1.57 (2). Further, the Rotterdam Study reported a greater risk of unruptured IA in females (odds ratio (OR), 1.92, 95% CI, 1.33–2.84) (9). Regarding IA rupture, a meta-analysis of individual patient data revealed that the female to male hazard ratio (HR) was 1.43 (95% CI, 1.07–1.93) (8). Another recent meta-analysis reported that aneurysmal SAH was more common in women than in men [HR, 1.90 (95% CI 1.47–2.46)] (10). Furthermore, Wang et al. reported a multicenter retrospective study in China, which also revealed that females had a greater risk of IA rupture [OR, 1.85 (95% CI, 1.05–3.39)] (11).

Nevertheless, controversy remains over whether sex is an independent risk factor for IA rupture (12–14). Studies using scores generated by a clinical IA rupture risk prediction tool (PHASE; population, hypertension, size of aneurysm, earlier SAH from another aneurysm, site of aneurysm), based on data from six cohort studies, revealed that sex has limited predictive value for IA rupture (15). Additionally, differences in the pathophysiology and anatomical features between the sexes are imprecise (16). Further, given the fundamental differences in morphology and size of IAs in various cerebral arteries, little is known about whether the size and location of IAs affect the risk of rupture in a sex-specific manner.

Therefore, exploring the sex disparities in the risk of IA rupture may provide useful knowledge in primary and secondary prevention of IA rupture. The aim of this study is to assess whether there are associations and differences in IA characteristics between female and male.

## 2 Methods

### 2.1 Study setting and data source

The study was set in two tertiary general hospitals. Data were obtained from electronic medical records during the period from 2012 to 2019.

This study was exempted from ethics committee approval for using retrospective and anonymized data with no further intervention involved. Informed consent was waived due to the retrospective nature of the study, and all private information was anonymized. The study conformed to the Declaration of Helsinki. This article follows the STROBE reporting guidelines (Supplementary material S2).

### 2.2 Participant inclusion and ascertainment of cases and controls

Patients with a clinical suspicion of IA, who subsequently underwent computed tomographic angiography (CTA) or digital subtraction angiography (DSA), were identified by searching the electronic medical and radiology records of the hospitals. Patients who had previously been diagnosed with IAs or had been treated at another hospital and undergone follow-up examination were excluded, with only the first examination in each patient’s medical procedure included in the study. Finally, patients who were diagnosed for positive IA with no previous IA record were included. The complete patient inclusion workflow is presented in Supplementary Figure S1. The process of image acquisition is described in the Supplementary material section.

A case–control study design was used to analyze the risk of the rupture, based on data from real world medical records. A case was defined as an original diagnosis of positive for IA rupture based on CTA and/or DSA, which was primarily made by one radiologist and one neurologist and then reviewed by one senior radiologist and one senior neurologist. Participants included in this study with un-rupture IA in the original diagnosis were selected as controls.

### 2.3 Ascertainment of IA characteristics

IA size and site were determined based on CTA and DSA results. All patients underwent non-contrast computed tomography prior to head and/or neck CTA. For patients with suspected aneurysmal SAH or intracerebral hemorrhage (ICH), DSA was performed at the discretion of the treating neurosurgeon.

Aneurysms were defined as rounded areas of contrast outpouching ≥2 mm in diameter. Patients with fusiform aneurysms were excluded from the analysis. Three-dimensional reconstructions of all CTA images were created in three planes, and maximum intensity pixel images of the intracranial internal carotid arteries, as well as the intracranial vertebral and basilar arteries, were also used for imaging interpretation. IAs were classified by location, size, and rupture status, based on the radiologist report and subsequent imaging review by two neuroradiologists. Aneurysm size was equal to the maximum aneurysm diameter. If a patient with SAH underwent both DSA and CTA, DSA measurements were used to report aneurysm site and size. For patients with multiple IAs, the ruptured aneurysms were identified based on imaging reports, and recorded separately in a patient level data and in a IA level data for further analysis.

Arteries of interest included: internal carotid artery (ICA), vertebral artery, basilar artery, anterior communicating artery (ACoA), posterior cerebral artery, middle cerebral artery (MCA), and anterior cerebral artery; aneurysms arising from other arteries were classified as “other arteries”.

### 2.4 Ascertainment of covariates

Several covariates were considered in the analysis, including age, IA size, IA, multiple IAs (yes vs. no), history of ICH (yes vs. no), history of ischemic stroke (yes vs. no), and hypertension (yes vs. no). Information was extracted from patient electronic medical records. Ascertainment of IA size and quantity is described above. Hypertension was determined based on preadmission medical records. A history of ICH or ischemic stroke was based on preadmission or outpatient medical inquiries by the attending physician.

### 2.5 Statistical analysis

Participant characteristics are summarized as proportions (%) for categorical variables, means and standard errors for normally distributed continuous variables, and medians ([Q1, Q3]) for non-normally distributed continuous variables. Categorical variables were compared using Fisher’s exact test, and non-normally distributed continuous variables were compared using the Kruskal-Wallis rank-sum test. Occasional missing values were omitted from all analyses. *p* < 0.05 (two-sided) was considered to indicate significance. Bonferroni correction was applied for multigroup pairwise comparisons.

Analyses of sex differences in IA size and artery location were performed at the lesion level. OR and 95% CI values from logistic regression analyses were used to assess associations between various factors and IA rupture. Spline regression was used to analyze the potential nonlinear association between IA size and IA rupture across age and artery subgroups in both sexes. ANOVA was used for nonlinear testing, and the cutoff for the restricted cubic spline was determined by the *R*^2^ value of each fitted model. Interactions between each subgroup and potential modifying factors (sex) were assessed using the likelihood ratio test. When continuous variables were converted to categorical variables, the lower boundary contained the value shown. E-value was used to evaluate the effect strength of potential unadjusted confounders and selection bias in subgroup analyses (17, 18). For sensitivity analysis, we also performed propensity score matching based on the collected IA characteristics to balance the differences between groups.

All the statistical analyses were performed using R software, version 4.2.2 (R Development Core Team, Vienna, Austria).

## 3 Results

### 3.1 Patient characteristics

A total of 1,883 IA patients were included in this study, with a total of 2,423 IAs identified. Among all IAs, 1,689 IAs were unruptured, and 734 IAs were ruptured (Table 1). The proportion of females was 59.3%, and patient age ranged from 12 to 93 years (mean, 61.2 ± 11.9 years). Most patients (91.9%) were 41–80 years old. The mean age of female patients was slightly older than that of male patients (61.7 ± 11.5 years vs. 60.5 ± 12.3 years, *p* = 0.034). There were no significant differences in hypertension, history of ICH, or history of ischemic stroke between females and males. Four-hundred-twenty (22.3%) patients were diagnosed with multiple aneurysms, and the proportion of female patients with multiple aneurysms was significantly higher than that of males (25.6% vs. 17.5%, *p* < 0.001). Additionally, there was a greater percentage of ruptured IAs in females (females, 42.5% vs. males, 33.8%, *p* < 0.001). At the lesion level (Supplementary Table S1), most IAs (50.6%) were located in the ICA, followed by the MCA (16.3%), and ACoA (15.3%). Overall IA location distribution differed between females and males; female IAs were more frequently located in the ICA than those in males (54.9% vs. 43.7%). Further, the mean diameter of ICAs with IAs in females was larger than that in males (6.60 vs. 5.50 mm).

**Table 1:** Basic characteristics of patients with IA.

Characteristic	Total (*n* = 1883)	Female (*n* = 1,117, 59.3%)	Male (*n* = 766, 40.7%)	*p*- value
Age, mean ± SD, years	61.19 ± 11.87	61.68 ± 11.5	60.48 ± 12.32	0.034
Age, *n* (%), years				0.105
≤40	69 (3.68)	37 (3.33)	32 (4.19)	
40–	295 (15.74)	161 (14.49)	134 (17.56)	
50–	498 (26.57)	289 (26.01)	209 (27.39)	
60–	584 (31.16)	355 (31.95)	229 (30.01)	
70–	345 (18.41)	223 (20.07)	122 (15.99)	
≥80	83 (4.43)	46 (4.14)	37 (4.85)	
Hypertension, *n* (%)	764 (40.55)	449 (40.16)	315 (41.12)	0.712
ICH history, *n* (%)	247 (13.11)	150 (13.42)	97 (12.66)	0.684
Ischemic stroke history, *n* (%)	476 (25.28)	275 (24.62)	201 (26.24)	0.459
IA rupture, *n* (%)	734 (38.96)	475 (42.49)	259 (33.81)	<0.001
Multiple IAs, *n* (%)				<0.001
Single	1,463 (77.71)	832 (74.42)	632 (82.51)	
Multiple (≥ 2)	420 (22.29)	286 (25.58)	134 (17.49)	

IA, intracranial aneurysm; ICH, intracerebral hemorrhage.

The proportions of smokers were 16.71% (128/766) and 0.5% (6/1117) among male and female patients, respectively, while and the proportions of smokers patients with unruptured and ruptured IAs were 11.2 and 4.1%, respectively; therefore, we decided to exclude smoking as covariable from our analyses, to avoid potential endogenous bias.

### 3.2 Comparison of IA characteristics between the ruptured and Unruptured groups according to sex

IA characteristics in the ruptured and unruptured groups according to sex are presented in Table 2; Supplementary Table S2. There was a greater proportion of females with IAs than males in both the unruptured (UIA) and ruptured (RIA) groups (*p* = 0.03). No significant difference in the age composition between sexes was detected in the UIA group; however, there was a significant difference in the age composition between sexes (*p* < 0.001) in the RIA group; females were older than males (60.29 ± 10.99 vs. 56.44 ± 10.69 years, *p* < 0.001).

**Table 2:** Basic characteristics of ruptured and unruptured IAs in female and male patients.

Characteristic		Overall			UIA			RIA	
	UIA (*n* = 1,689)	RIA (*n* = 734)	*p*- value	Female (*n* = 1,014)	Male (*n* = 675)	*p* -value	Female (*n* = 475)	Male (*n* = 259)	*p*- value
Sex, *n* (%)			0.033			<0.001			<0.001
Female	1,014 (60.04)	475 (64.71)		1,014			475		
Male	675 (39.96)	259 (35.29)			675			259	
Age, mean ± SD, years	63.12 ± 11.96	58.93 ± 11.03	<0.001	63.40 ± 11.54	62.71 ± 12.55	0.252	60.29 ± 10.99	56.44 ± 10.69	<0.001
Age, *n* (%), years			<0.001			0.322			<0.001
≤ 40	48 (2.86)	33 (4.50)		26 (2.58)	22 (3.27)		19 (4.00)	14 (5.41)	
40–	207 (12.34)	137 (18.66)		112 (11.13)	95 (14.14)		79 (16.63)	58 (22.39)	
50–	391 (23.30)	232 (31.61)		238 (23.66)	153 (22.77)		132 (27.79)	100 (38.61)	
60–	540 (32.18)	222 (30.25)		328 (32.60)	212 (31.55)		157 (33.05)	65 (25.10)	
70–	397 (23.66)	94 (12.81)		249 (24.75)	148 (22.02)		75 (15.79)	19 (7.34)	
≥ 80	95 (5.66)	16 (2.18)		53 (5.27)	42 (6.25)		13 (2.74)	3 (1.16)	
Artery of IA, *n* (%)			<0.001			0.002			<0.001
ICA	932 (55.18)	294 (40.05)		593 (58.48)	339 (50.22)		225 (47.37)	69 (26.64)	
VA	51 (3.02)	23 (3.13)		25 (2.47)	26 (3.85)		15 (3.16)	8 (3.09)	
BA	67 (3.97)	11 (1.50)		34 (3.35)	33 (4.89)		6 (1.26)	5 (1.93)	
ACoA	169 (10.01)	204 (27.79)		80 (7.89)	89 (13.19)		99 (20.84)	105 (40.54)	
ACA	99 (5.86)	50 (6.81)		61 (6.02)	38 (5.63)		31 (6.53)	19 (7.34)	
MCA	270 (15.99)	126 (17.17)		161 (15.88)	109 (16.15)		78 (16.42)	48 (18.53)	
PCA	56 (3.32)	10 (1.36)		36 (3.55)	20 (2.96)		7 (1.47)	3 (1.16)	
Other arteries	45 (2.66)	16 (2.18)		24 (2.37)	21 (3.11)		14 (2.95)	2 (0.77)	
Size, mean ± SD, mm	7.90 ± 6.28	7.86 ± 3.97	0.849	7.96 ± 6.19	7.81 ± 6.42	0.645	7.88 ± 4.03	7.82 ± 3.87	0.867
Size, n (%), mm			<0.001			0.741			0.752
≤ 3	104 (6.16)	10 (1.36)		67 (6.61)	37 (5.48)		8 (1.68)	2 (0.77)	
3–	524 (31.02)	140 (19.07)		305 (30.08)	219 (32.44)		86 (18.11)	54 (20.85)	
5–	389 (23.03)	220 (29.97)		234 (23.08)	155 (22.96)		141 (29.68)	79 (30.50)	
7–	234 (13.85)	169 (23.02)		139 (13.71)	95 (14.07)		112 (23.58)	57 (22.01)	
9–	153 (9.06)	95 (12.94)		100 (9.86)	53 (7.85)		66 (13.89)	29 (11.20)	
11–	83 (4.91)	48 (6.54)		50 (4.93)	33 (4.89)		31 (6.53)	17 (6.56)	
≥ 13	202 (11.96)	52 (7.08)		119 (11.74)	83 (12.30)		31 (6.53)	21 (8.11)	
Multiple IA, *n* (%)	813 (48.13)	147 (20.03)	<0.001	539 (53.16)	274 (40.59)	< 0.001	119 (25.05)	28 (10.81)	<0.001
Hypertension, *n* (%)	583 (34.52)	417 (56.81)	<0.001	351 (34.62)	232 (34.37)	0.959	263 (55.37)	154 (59.46)	0.322
ICH history, *n* (%)	147 (8.70)	170 (23.16)	<0.001	86 (8.48)	61 (9.04)	0.757	114 (24.00)	56 (21.62)	0.523
Ischemic stroke, *n* (%)	515 (30.55)	134 (18.26)	<0.001	298 (29.48)	217 (32.15)	0.266	93 (19.58)	41 (15.83)	0.248

IA, intracranial aneurysm; RIA, ruptured intracranial aneurysm; UIA, unruptured intracranial aneurysm; ICH, intracerebral hemorrhage; ICA, internal carotid artery; VA, vertebral artery; BA, basilar artery; ACoA, anterior communicating artery; ACA, anterior cerebral artery; MCA, middle cerebral artery; PCA, posterior cerebral artery.

When comparing sex differences between IAs from patients who had single and multiple IAs, we found there was a higher aneurysm rupture proportion for female than male patients in both single and multiple IA groups (42.8% vs. 36.5% in the single IA group and 18.1% vs. 9.3% in the multiple IA group, Supplementary Table S6).

The ICA was the most common site of aneurysm in both females (58.48%) and males (50.22%), while in the RIA group, the ICA (47.37%) was the most frequent location in females, while the ACoA (40.5%) was the most frequent location in males.

Regarding aneurysm size, 3–7 mm was most common, accounting for 67.91 and 72.06% of UIA and RIA group aneurysms, respectively. More giant aneurysms (≥ 13 mm) were recorded in the UIA group than in the RIA group (11.96% vs. 7.08%); however, when grouped by sex, aneurysm size distributions were similar in the UIA and RIA groups. Multiple IAs were more frequent in the UIA group and in females. A history of hypertension and intracerebral hemorrhage was more common in the UIA group, while ischemic stroke was more common in the RIA group; however, comparisons between the RIA and UIA groups did not reveal significant associations of sex with the risk of hypertension, intracerebral hemorrhage, or ischemic stroke.

**Figure 1:**
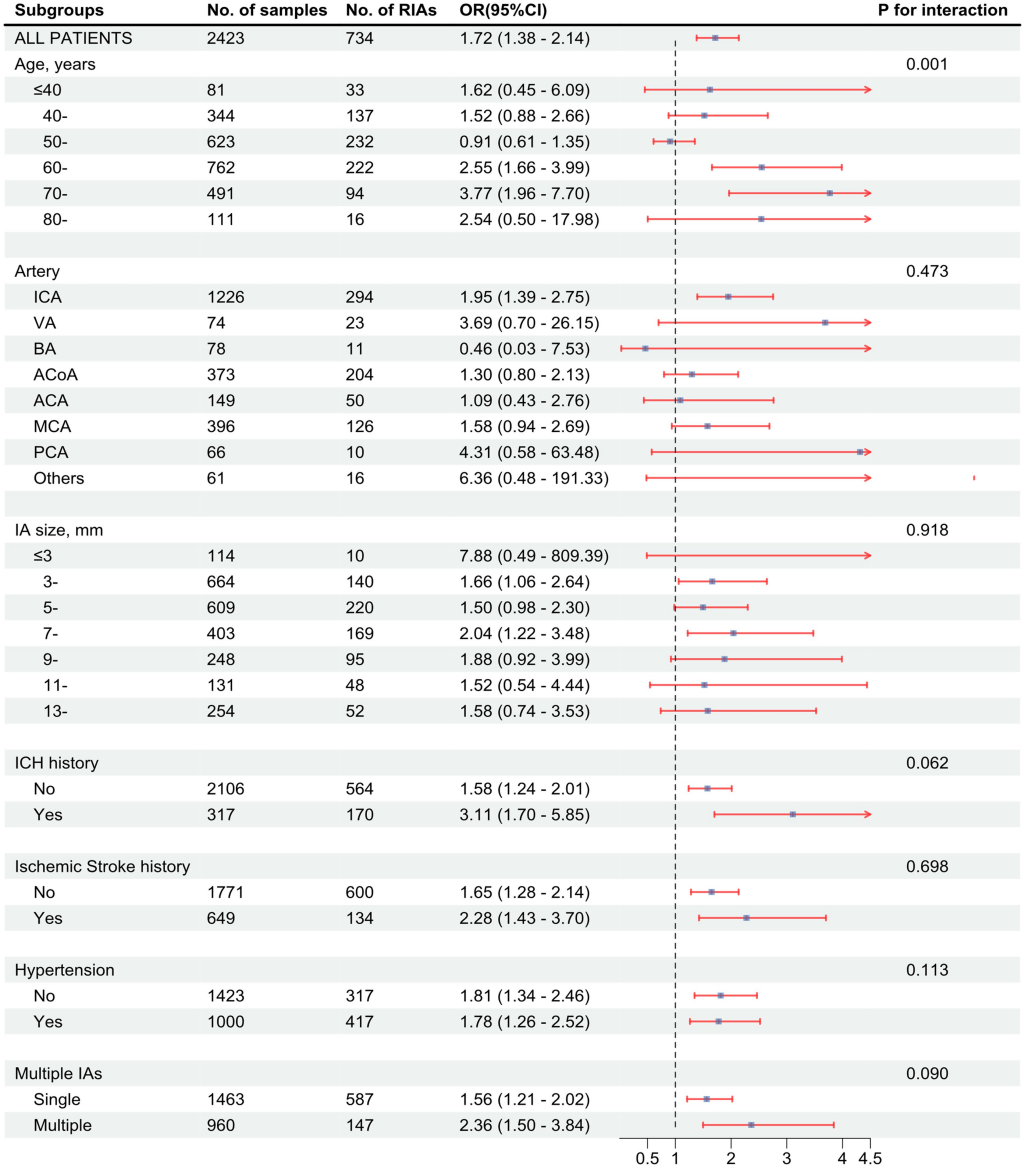
Associations between being female sex and intracranial aneurysm (IA) rupture in subgroups. Forest plot showing odds ratio (OR) with 95% confidence interval (95% CI) values of ruptured intracranial aneurysms (RIAs) in females compared with males. *p* value for interaction was used to analyze whether there was an effect modification across the different subgroups. OR values were calculated based on a logistic model adjusted for age, artery location, IA size, history of intracerebral hemorrhage (ICH), history of ischemic stroke, hypertension, and IA multiplicity. The *p* value for interaction is based on an ANOVA of interaction terms within the model used. ICA, internal carotid artery. VA, vertebral artery; BA, basilar artery; ACoA, anterior communicating artery; ACA, anterior cerebral artery; MCA, middle cerebral artery; PCA, posterior cerebral artery.

**Figure 2:**
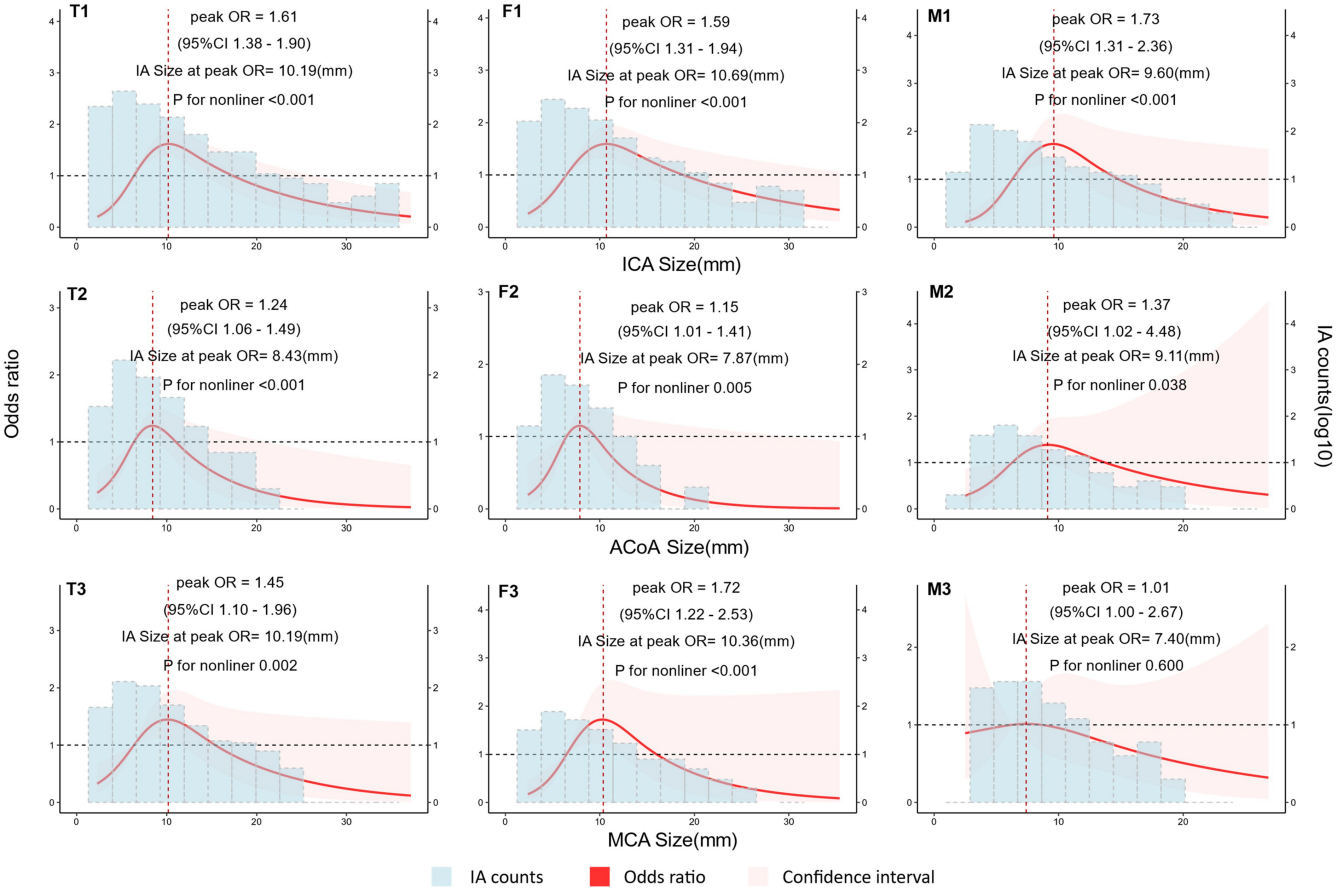
Nonlinear association between intracranial aneurysm (IA) size and rupture according to sex. Plot showing the association of IA size with IA rupture across different arteries according to sex. An n-shaped association was found for the ICA, ACoA, and MCA in females and for the ICA in males. Odds ratio (OR) and 95% confidence interval (95% CI) values were calculated per unit (mm) change using restricted cubic spline analysis, then adjusted for history of intracerebral hemorrhage, history of ischemic stroke, hypertension, IA multiplicity, and age. OR changed with IA size. IA count density is displayed on a log10 scale. T1, total IAs in the internal carotid artery (ICA); T2, total IAs in the anterior communicating artery (ACoA); T3, total IAs in the middle cerebral artery (MCA); F1, IAs in the ICA of females; F2, IAs in the ACoA of females; F3, IAs in the MCA of females; M1, IAs in the ICA of males; M2, IAs in the ACoA of males; M3, IAs in the MCA of males.

**Figure 3:**
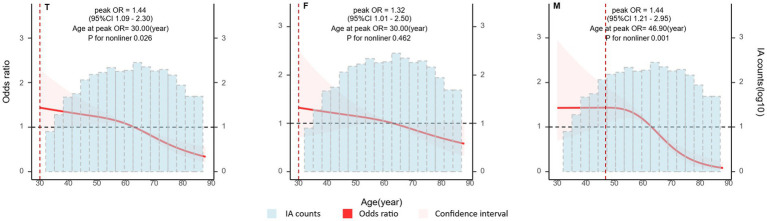
Associations between patient age and intracranial aneurysm (IA) rupture by sex. Plot showing the association between patient age and IA rupture. A nonlinear association was detected in males. Odds ratio (OR) and 95% confidence interval (CI) values were calculated per unit (year) change using restricted cubic spline analysis, then adjusted for history of intracerebral hemorrhage, history of ischemic stroke, hypertension, IA multiplicity, and IA size. IA, intracranial aneurysm; T, both sexes; F, female group; M, male group.

The risk of aneurysm rupture in females compared with that in males across each subgroup is illustrated in Figure 1. Overall, the risk of rupture in females was greater [adjusted OR (aOR), 1.72 (95% CI, 1.38–2.14)], regardless of history of stroke, hypertension, or multiple aneurysms. Across age groups, the risk of rupture was only greater in females in the 60–70 and 70–80 year-old groups [aOR, 2.55 (95% CI, 1.66–3.99) and 3.77 (95% CI, 01.96–7.70), respectively]. There was a greater risk of ruptured aneurysm in the ICA in females [aOR, 1.95 (95% CI, 1.39–2.75)] than that in males. In the subgroups based on the covariates used in this study, we only identified statistically significant interaction between sex and age. In the results of propensity score matching, we found that females have a higher risk of IA rupture [aOR, 1.57 (95% CI, 1.25–1.99), Supplementary Table S5], which is similar to the result of direct grouping. A comparison of risk factors for ruptured aneurysm between females and males is presented in Table 3. N-shaped associations between IA size and the risk of rupture in both sexes were similar, with IAs 7–11 mm in size at greater risk of rupture (Figure 2; Supplementary Figure S2). Spline regression analysis showed that the association between age and aneurysm rupture was linear in females, while that in males was nonlinear. Risk of rupture was highest in females aged 30 years and decreased with age. There was no age difference in men until 50 years old, after which the risk decreased with age (Figure 3; Supplementary Table S2). E value calculation indicated that the results were robust and without confounding bias (Supplementary Figure S4).

**Table 3:** Associations between risk factors and intracranial aneurysm rupture by sex.

Variable	Total		Female		Male	
	aOR (95% CI)*	*p* for trend	aOR (95% CI)*	*p* for trend	aOR (95% CI)*	*p* for trend
Female Sex	1.72 (1.38–2.14)					
Age, years		<0.001		0.012		<0.001
≤40	1.83 (1.06–3.14)		1.80 (0.86–3.73)		2.32 (0.99–5.41)	
4–	1.51 (1.10–2.06)		1.28 (0.85–1.91)		2.07 (1.23–3.50)	
50–	1.27 (0.97–1.65)		0.92 (0.66–1.29)		2.34 (1.48–3.73)	
60–	1.00 (reference)		1.00 (reference)		1.00 (reference)	
70–	0.66 (0.48–0.90)		0.73 (0.51–1.06)		0.44 (0.23–0.83)	
≥80	0.48 (0.25–0.86)		0.54 (0.26–1.06)		0.29 (0.06–0.94)	
Artery of IA						
ICA	1.00 (reference)		1.00 (reference)		1.00 (reference)	
VA	1.36 (0.75–2.38)		1.63 (0.77–3.37)		1.10 (0.41–2.76)	
BA	0.51 (0.23–1.02)		0.47 (0.16–1.16)		0.59 (0.17–1.79)	
ACoA	2.73 (2.05–3.63)		2.43 (1.67–3.56)		3.19 (2.02–5.07)	
ACA	1.09 (0.71–1.65)		0.91 (0.54–1.53)		1.64 (0.77–3.42)	
MCA	1.17 (0.87–1.55)		1.08 (0.75–1.54)		1.45 (0.87–2.40)	
PCA	0.51 (0.22–1.08)		0.48 (0.17–1.19)		0.53 (0.11–1.87)	
Other arteries	1.06 (0.53–2.02)		1.45 (0.65–3.12)		0.43 (0.06–1.80)	
Size, mm		0.510		0.518		0.863
≤ 3	0.35 (0.16–0.71)		0.42 (0.17–0.95)		0.27 (0.04–1.05)	
3–	1.00 (reference)		1.00 (reference)		1.00 (reference)	
5–	1.85 (1.39–2.48)		1.80 (1.25–2.59)		2.17 (1.32–3.61)	
7–	2.55 (1.86–3.50)		2.68 (1.81–3.97)		2.37 (1.36–4.14)	
9–	2.26 (1.56–3.26)		2.37 (1.52–3.70)		2.36 (1.21–4.58)	
11–	2.05 (1.29–3.25)		2.25 (1.27–3.97)		1.78 (0.77–4.06)	
≥13	1.09 (0.72–1.62)		1.07 (0.64–1.78)		1.10 (0.55–2.14)	
Multiple IA		<0.001		<0.001		<0.001
Single	1.00 (reference)		1.00 (reference)		1.00 (reference)	
Multiple	0.27 (0.21–0.34)		0.29 (0.22–0.39)		0.18 (0.11–0.29)	
ICH history	3.21 (2.41–4.29)		4.00 (2.79–5.77)		2.33 (1.42–3.83)	
Ischemic stroke	0.39 (0.29–0.51)		0.43 (0.30–0.60)		0.31 (0.19–0.50)	
Hypertension	4.32 (3.44–5.44)		3.74 (2.82–4.98)		5.83 (3.92–8.81)	

*Adjusted for IA in other arteries, history of intracerebral hemorrhage, history of ischemic stroke, hypertension, age, size of IA, and sex. This model is fitted at the patient level for corrected adjustment of the artery variable. The reference level was set based on which level had the largest size in the study sample. IA, intracranial aneurysm; aOR, adjusted odds ratio; CI, confidence interval; ICA, internal carotid artery; VA, vertebral artery; BA, basilar artery; ACoA, anterior communicating artery; ACA, anterior cerebral artery; MCA, middle cerebral artery; PCA, posterior cerebral artery.

## 4 Discussion

In this study, we explored sex-specific factors associated with IAs and ruptured IAs in a Chinese population. To the best of our knowledge, there have been no similar reports to date. Almost 60% of patients with IAs were female, and the rate of IA rupture was greater in females than that in males. These results are consistent with previous reports (6–8).

The main risk factors for aneurysm development and rupture risk are considered to include genetic factors, age, hypertension, behavioral factors, such as smoking and alcohol consumption, estrogen deficiency, aneurysm size, morphology, and location, and blood flow pressure factors (12, 15, 19–21). Although women have a greater risk of aneurysm rupture than men, this sex difference cannot be explained by differences in risk factors established as associated with aneurysm occurrence and rupture (20). Therefore, more studies are needed to explore the factors influencing sex differences in aneurysm occurrence and rupture.

Our findings regarding aneurysm location are similar to those of previous reports, showing that IAs occur predominantly in the ICA, ACoA, and MCA (4, 6, 22); however, most previous studies did not explore sex differences in aneurysm sites. In the present study, we found that ICA aneurysms were more common in females, whereas ACoA aneurysms were more common in males, similar to a previous report from Japan (23). Nevertheless, after controlling for covariates, we found that the highest risk of IA rupture in both females and males was in the ACoA (Table 3). Another study that included a Chinese population of 2,085 patients with SAH also showed that the predominant aneurysm rupture site in both men and women was the ACoA (24). These findings suggest that aneurysm site is not a sex-specific factor for the risk of aneurysm rupture.

Many studies have shown a positive correlation between IA size and risk of rupture, with larger aneurysms having a greater risk of rupture (7, 25–27); based on this, assessment methods, such as the HASE score, have also proposed aneurysm size as a threshold for assessing risk of rupture (15). In this study, we grouped the arteries with higher proportions of aneurysms into three subgroups (ICA, ACoA, and MCA) and compared the sex differences between aneurysm size and rupture risk in each subgroup separately. We also divided patients into 40–49, 50–59, 60–69, and 70–79 year-old age groups, and compared the sex differences between aneurysm size and rupture risk separately. We found similar “n”-shaped associations between aneurysm size and rupture for the different artery and age subgroups in both sexes, after controlling for other covariates (Figure 2; Supplementary Figure S2), suggesting that aneurysm size is not an influential factor contributing to the sex difference in aneurysm rupture.

Our univariate analyses indicated that factors associated with rupture included age, aneurysm site, aneurysm size, multiple aneurysms, hypertension, and stroke, when sex was not taken into account (Table 2), similar to previous reports (8, 28). In multivariate analyses, age, aneurysm size, and location were associated with rupture; however, when grouped by sex, the only factor with sex-differentiated effects was age. Numerous studies have identified age differences in the incidence of cerebral aneurysms in men and women [40], but few have compared the age distribution of IA ruptures by sex. We found that IA rupture occurred approximately 10 years earlier in males than in females, with a greater incidence in the 50–59 years age group in males and in the 60–69 years age group in females. These results are consistent with those of a previous study showing that IA ruptures occur earlier in males than in females (29). Since IA rupture is more common in women older than 50 years, many researchers have proposed that the development of cerebral aneurysms is associated with estrogen deficiency (28, 30–32). We found that the frequency of IAs was greater in females than males in every age group (Table 2), but the age distributions of UIAs in females and males were identical and normally distributed, with the highest prevalence in the 50–70-year-old group. If estrogen deficiency is a major factor in aneurysm development, it is unlikely to show this same age distribution in both sexes. Therefore, to assess whether menopause can account for the difference in the risk of IA rupture between women and men, we analyzed the relationship between age and the risk of IA rupture by sex, using nonlinear correlation analysis after controlling for the relevant covariates. We found that the risk of IA rupture did not rise with increasing age in either sex (Figure 3). Rather, risk of IA rupture was greater in younger than older patients with aneurysms. Notably, risk of IA rupture was highest in females at 30 years old and males aged 30–50 years, and tended to decrease with age in both sexes. Our results are similar to those of a previous cohort study in Canada (33). This feature of IA rupture differs from observations of non-aneurysmal SAH, risk of which increases with age (34). This result indirectly suggests that estrogen deficiency is not an independent risk factor for IA rupture. A meta-analysis showed that hormone replacement therapy use is associated with increased rates of any stroke and cause-specific stroke, including ischemic stroke and SAH (35). Additionally, a previous study reported that serum estradiol levels were not associated with the occurrence or size of cerebral aneurysms (6). Therefore, the relationship between estrogen deficiency and the development and rupture of IAs warrants further study, and estrogen deficiency may not be an independent risk factor for IA.

Hypertension is a relatively well-defined risk factor for IA rupture, as confirmed in the present study. The incidence of hypertension is greater in men than that in women up to the age of 60 years; however, after 60 years, the prevalence of hypertension is significantly greater in women than men (36, 37). Moreover, women have lower rates of blood pressure control than men after 65 years old (38). In particular, systolic blood pressure levels and pulse pressure differentials are significantly greater in women than men after 60 years old (38). This factor has also recently been suggested to increase vascular pressure through hemodynamic load-induced elastic fiber degradation and collagen deposition in the arterial wall, thereby increasing the risk of hemorrhagic stroke (36). Recently, hemorrhagic stroke has declined globally, in line with the trend in the control of hypertension (particularly systolic blood pressure) following the implementation of measures to strengthen hypertension control (12). In this study, the age distribution of IA rupture by sex was highly consistent with the age distribution of hypertension by sex, suggesting that elevated blood pressure, particularly systolic blood pressure, is the most important factor in IA rupture. Blood pressure control is an important measure for preventing IA rupture, especially in women.

IA frequency was greater in females than in males in all age groups, but the distribution pattern by age was consistent in females and males, suggesting the importance of genetic factors. Previous studies have shown that a number of monogenic and polygenic genetic disorders are associated with IA (21). Physiological sex differences attributable to genetic factors may also be responsible for sex differences in the occurrence of IAs; for example, the anatomic difference in the circle of Willis between females and males (28).

### 4.1 Strengths and limitations

This study had a number of strengths. First, we used real-world data and included a representative sample of all patients with IA from two tertiary general hospitals over eight consecutive years for analysis. Second, the study population included only patients undergoing first diagnosis, which may have prevented survival bias. Third, we used nonlinear correlation analysis to analyze the risk factors associated with IA rupture, whereas most previous studies conducted linear correlation analyses; our approach allowed us to determine that age tended to be negatively correlated with the risk of rupture. Fourth, we conducted subgroup analyses by sex. Our study also had several limitations. First, due to the hospital privacy policy, we were unable to obtain all raw data, such as CTA 3D reconstruction data; therefore, we were unable to include analyses of IA shape, aspect ratio, size ratio, or bottleneck factor aspect ratio in our study. Second, as our data were obtained from patients attending hospitals, asymptomatic patients and those who died before admission were not included. Third, as this study utilized data from real-world clinical practice, certain factors such as living environmental factors and genetic factors that were not investigated during the clinical process could not be analyzed in the study.

## 5 Conclusion

Our study provides evidence that females have a greater prevalence of IA and a higher rate of rupture than males, and that genetic factors, as well as hypertension, may be important factors underlying these differences. We hypothesize that the high incidence of IA in women may be related to inheritance of X-chromosome-linked factors, rather than estrogen deficiency, and that the sex difference in the risk of aneurysm rupture is attributable to age and hypertension; however, our conclusions should be interpreted with caution, as this was an observational study based on data from medical records. Further basic and epidemiological studies are needed to assess sex differences in IA formation, growth mechanisms, and rupture.

## Data Availability

The raw data supporting the conclusions of this article will be made available by the authors, without undue reservation.
