# Supplementary material for: Sex disparities in the risk of intracranial aneurysm rupture: a case–control study
Source: Front Neurol. 2024 Dec 27;15:1483679. doi: 10.3389/fneur.2024.1483679 (PMC11720961; doi:10.3389/fneur.2024.1483679)

[Supplementary method 2](#_Toc183707258)

[Image acquisition 2](#_Toc183707259)

[Supplementary results 3](#_Toc183707260)

[Supplementary Table 1. Artery location and size of intracranial aneurysms in different sex at aneurysm level 3](#_Toc183707261)

[Supplementary Table 2. The risk of rupture intracranial aneurysms by age (per unit, year) 4](#_Toc183707262)

[Supplementary Table 3. The risk of rupture intracranial aneurysms by size (per unit, mm) 5](#_Toc183707263)

[Supplementary Table 4. E-value and Selection E-value on association of female sex and intracranial aneurysm rupture 6](#_Toc183707264)

[Supplementary Figure 1. Flowchart of patient inclusion 8](#_Toc183707265)

[Supplementary Figure 2. Non-linear association between size of intracranial aneurysms and rupture by age in different sex 9](#_Toc183707266)

[Supplementary Figure 3. Pairwise comparisons of intracranial aneurysm size across each artery 10](#_Toc183707267)

[Supplementary Figure 4. E-value plot of association between female sex and intracranial aneurysm rupture 11](#_Toc183707268)

[Supplementary Figure 5. Change in effect estimate of female sex of different confounding variables 12](#_Toc183707269)

## Supplementary method

## Image acquisition

The medical institutes of this study used a 128-slice CT scanner, with the tube voltage set at 100–120 kV and the effective tube current at 150–200 mAs, or automatic tube current modulation technology. Parameters were adjusted according to the patient's condition in special cases. The rotation speed was 0.27–0.5 s/r, with a pitch of 1.0. The display field of view was 226 × 240 mm. The slice thickness was 0.5 mm, the reconstruction interval was 0.5 mm, the reconstruction matrix was 512 × 512, and the collimator width was set to the widest CTA scans of the head and neck were performed via intravenous group injection, an iodine contrast concentration of 370–400 mg/ml, an automatic threshold trigger for scanning, a flow rate of 4.5–5.0 ml/s, and a dose of 1.5–2.0 ml/kg body weight. Scanning ranged from the top of the skull to the level of the aortic arch, including the aortic arch and the opening of the three arteries.

## Supplementary results

## Supplementary Table 1. Artery location and size of intracranial aneurysms in different sex at aneurysm level

| Variables | Total（N = 2423） | Sexes | | |
| --- | --- | --- | --- | --- |
|  |  | Female（N = 1489） | Male（N_IA_ = 934） | *P* value |
| Artery location, n(%) |  |  |  | <0.001 |
| ICA | 1226 (50.60) | 818 (54.94) | 408 (43.68) |  |
| VA | 74 (3.05) | 40 (2.69) | 34 (3.64) |  |
| BA | 78 (3.22) | 40 (2.69) | 38 (4.07) |  |
| ACoA | 373 (15.39) | 179 (12.02) | 194 (20.77) |  |
| ACA | 149 (6.15) | 92 (6.18) | 57 (6.10) |  |
| MCA | 396 (16.34) | 239 (16.05) | 157 (16.81) |  |
| PCA | 66 (2.72) | 43 (2.89) | 23 (2.46) |  |
| Others | 61 (2.52) | 38 (2.55) | 23 (2.46) |  |
| Size, median [Q1,Q3], mm | 6.40 [4.60, 9.20] | 6.50 [4.60, 9.30] | 6.30 [4.53, 9.10] | 0.503 |
| ICA | 6.30 [4.40, 9.30] | 6.60 [4.60, 9.50] | 5.50 [4.10, 8.22] | <0.001 |
| VA | 7.70 [5.90, 10.28] | 7.00 [4.68, 8.85] | 8.40 [7.05, 12.05] | 0.015 |
| BA | 8.60 [5.35, 12.80] | 7.20 [4.75, 11.50] | 10.75 [6.12, 13.92] | 0.082 |
| ACoA | 6.50 [5.10, 8.80] | 6.40 [5.10, 8.60] | 6.55 [5.10, 8.88] | 0.513 |
| ACA | 5.90 [4.30, 7.80] | 5.90 [4.38, 7.30] | 5.90 [4.30, 8.00] | 0.986 |
| MCA | 7.10 [4.97, 10.03] | 6.80 [4.70, 10.05] | 7.30 [5.20, 10.00] | 0.201 |
| PCA | 5.45 [4.30, 7.18] | 5.50 [4.30, 6.55] | 5.40 [4.50, 8.10] | 0.637 |
| Others | 4.80 [3.90, 6.40] | 4.75 [4.10, 5.90] | 5.00 [3.50, 6.95] | 0.970 |

BA, Basilar artery. VA, Vertebral artery. ACoA, Anterior Communicating Artery. PCA, Posterior Cerebral Artery. MCA, Middle Cerebral Artery. ACA, Anterior Cerebral Artery. ICA, Internal Carotid Artery.

## Supplementary Table 2. The risk of rupture intracranial aneurysms by age (per unit, year)

| Artery | Total |  | Female |  | Male |  |
| --- | --- | --- | --- | --- | --- | --- |
|  | OR (95%CI) | *P* value for non-linear | OR (95%CI) | *P* value for non-linear | OR (95%CI) | *P* value for non-linear |
| ICA | 0.98(0.97-0.99) | 0.444 | 0.99(0.97-1.00) | 0.942 | 0.95(0.93-0.98) | 0.032 |
| VA | 0.95(0.89-1.01) | 0.256 | 0.95(0.86-1.02) | 0.258 | 0.96(0.82-1.09) | 0.596 |
| BA | 0.92(0.83-0.99) | 0.155 | 0.94(0.74-1.12) | 0.277 | 0.88(0.72-0.98) | 0.797 |
| ACoA | 0.99(0.97-1.01) | 0.314 | 0.99(0.95-1.02) | 0.261 | 0.98(0.95-1.01) | 0.045 |
| ACA | 1.00(0.96-1.03) | 0.649 | 1.00(0.96-1.05) | 0.760 | 0.97(0.90-1.03) | 0.956 |
| MCA | 0.96(0.93-0.98) | 0.125 | 0.97(0.94-1.00) | 0.201 | 0.92(0.88-0.96) | 0.087 |
| PCA | 1.01(0.95-1.08) | 0.295 | 1.08(0.98-1.23) | 0.521 | 0.78(0.42-1.02) | 0.332 |
| Others | 0.97(0.90-1.03) | 0.208 | 0.96(0.88-1.03) | 0.082 | 788722.76(0.00-Inf) | 0.849 |

OR, odds ratio. CI, confidence interval. BA, Basilar artery. VA, Vertebral artery. ACoA, Anterior Communicating Artery. PCA, Posterior Cerebral Artery. MCA, Middle Cerebral Artery. ACA, Anterior Cerebral Artery. ICA, Internal Carotid Artery.

## Supplementary Table 3. The risk of rupture intracranial aneurysms by size (per unit, mm)

| Artery | Overall |  | Female |  | Male |  |
| --- | --- | --- | --- | --- | --- | --- |
|  | OR (95%CI) | *P* value for non-linear | OR (95%CI) | *P* value for non-linear | OR (95%CI) | *P* value for non-linear |
| ICA | 1.01(0.99-1.04) | <0.001 | 1.01(0.98-1.04) | <0.001 | 1.05(0.99-1.11) | <0.001 |
| VA | 0.86(0.71-1.00) | 0.755 | 0.82(0.61-1.01) | 0.225 | 0.59(0.19-1.01) | 0.106 |
| BA | 0.76(0.50-0.97) | 0.355 | 0.67(0.28-1.05) | 0.519 | 0.71(0.35-1.00) | 0.409 |
| ACoA | 1.02(0.95-1.09) | <0.001 | 1.01(0.91-1.12) | 0.005 | 1.04(0.95-1.13) | 0.038 |
| ACA | 1.03(0.91-1.17) | 0.058 | 1.05(0.90-1.23) | 0.075 | 0.94(0.71-1.20) | 0.458 |
| MCA | 1.00(0.96-1.05) | 0.002 | 1.03(0.97-1.10) | <0.001 | 0.96(0.87-1.03) | 0.600 |
| PCA | 0.97(0.72-1.21) | 0.068 | 0.98(0.67-1.35) | 0.142 | 0.81(0.45-1.17) | 0.748 |
| Others | 0.90(0.63-1.13) | 0.380 | 0.94(0.65-1.25) | 0.174 | 8.52(0.00-INF) | 0.959 |

OR, odds ratio. CI, confidence interval. BA, Basilar artery. VA, Vertebral artery. ACoA, Anterior Communicating Artery. PCA, Posterior Cerebral Artery. MCA, Middle Cerebral Artery. ACA, Anterior Cerebral Artery. ICA, Internal Carotid Artery.

## Supplementary Table 4. E-value and Selection E-value on association of female sex and intracranial aneurysm rupture

| Subgroups | Crude OR of female sex(95%CI) | | E-value of female sex (95%CI) | | Selection E-value of female sex (95%CI) |
| --- | --- | --- | --- | --- | --- |
| ALL PATIENTS | 1.22(1.02-1.46) | | 2.83(2.11-) | | 1.95(1.63-) |
| Age, years |  | |  | |  |
| ≤40 | 1.15(0.47-2.83) | | 2.62(1.00-) | | 1.86(1.00-) |
| 40- | 1.16(0.75-1.79) | | 2.41(1.00-) | | 1.77(1.00-) |
| 50- | 0.85(0.61-1.18) | | 1.44(-1.00) | | 1.28(-1.00) |
| 60- | 1.56(1.12-2.20) | | 4.54(2.70-) | | 2.57(1.90-) |
| 70- | 2.35(1.39-4.14) | | 7.00(3.34-) | | 3.29(2.15-) |
| 80- | 3.43(1.03-15.69) | | 4.52(1.00-) | | 2.57(1.00-) |
| Artery |  | |  | |  |
| ICA | 1.86(1.39-2.53) | | 3.31(2.13-) | | 2.14(1.64-) |
| VA | 1.95(0.72-5.60) | | 6.84(1.00-) | | 3.25(1.00-) |
| BA | 1.16(0.32-4.39) | | 3.75(-1.00) | | 2.30(-1.00) |
| ACoA | 1.05(0.70-1.58) | | 1.92(1.00-) | | 1.54(1.00-) |
| ACA | 1.02(0.51-2.07) | | 1.40(1.00-) | | 1.26(1.00-) |
| MCA | 1.10(0.71-1.70) | | 2.53(1.00-) | | 1.82(1.00-) |
| PCA | 1.30(0.32-6.53) | | 8.09(1.00-) | | 3.57(1.00-) |
| Others | 6.12(1.48-41.95) | | 12.20(1.00-) | | 4.48(1.00-) |
| IA size |  | |  | |  |
| ≤3 | 2.21(0.52-15.15) | | 15.24(1.00-) | | 5.06(1.00-) |
| 3- | 1.14(0.78-1.68) | | 2.71(1.31-) | | 1.90(1.20-) |
| 5- | 1.18(0.84-1.67) | | 2.36(1.00-) | | 1.75(1.00-) |
| 7- | 1.34(0.89-2.03) | | 3.50(1.74-) | | 2.21(1.45-) |
| 9- | 1.21(0.70-2.10) | | 3.17(1.00-) | | 2.09(1.00-) |
| 11- | 1.20(0.58-2.54) | | 2.41(1.00-) | | 1.77(1.00-) |
| 13- | 1.03(0.56-1.94) | | 2.54(1.00-) | | 1.83(1.00-) |
| ICH history |  | |  | |  |
| No | 1.18(0.96-1.44) | | 2.53(1.78-) | | 1.82(1.47-) |
| Yes | 1.44(0.91-2.29) | | 5.67(2.79-) | | 2.92(1.93-) |
| Ischemic Stroke history | |  | |  | |
| No | 1.13(0.92-1.38) | | 2.69(1.88-) | | 1.89(1.52-) |
| Yes | 1.65(1.11-2.50) | | 3.98(2.20-) | | 2.38(1.67-) |
| Hypertension | |  | |  | |
| No | 1.35(1.04-1.76) | | 3.03(2.02-) | | 2.03(1.59-) |
| Yes | 1.13(0.87-1.46) | | 2.95(1.84-) | | 2.00(1.49-) |
| Multiple IAs |  | |  | |  |
| Single | 1.30(1.05-1.61) | | 2.50(1.72-) | | 1.81(1.43-) |
| Multiple | 2.16(1.42-3.40) | | 4.16(2.36-) | | 2.45(1.75-) |

OR, odds ratio. CI, confidence interval. BA, Basilar artery. VA, Vertebral artery. ACoA, Anterior Communicating Artery. PCA, Posterior Cerebral Artery. MCA, Middle Cerebral Artery. ACA, Anterior Cerebral Artery. ICA, Internal Carotid Artery.

## Supplementary Table 5. Associations between risk factors and intracranial aneurysm rupture based on propensity matching data.

| Variable |  | Total | |  | Female | |  | Male | |
| --- | --- | --- | --- | --- | --- | --- | --- | --- | --- |
|  |  | **aOR (95% CI)*** | ***P* for trend** |  | **aOR (95% CI)*** | ***P* for trend** |  | **aOR (95% CI)*** | ***P* for trend** |
| Female Sex |  | 1.57 (1.25-1.99) |  |  |  |  |  |  |  |
| Age, years |  |  | 0.162 |  |  | 0.284 |  |  | < 0.001 |
| ≤ 40 |  | 1.67 (0.92-3.08) |  |  | 1.51 (0.68-3.52) |  |  | 2.70 (1.06-7.18) |  |
| 4– |  | 1.26 (0.91-1.74) |  |  | 1.00 (0.66-1.53) |  |  | 1.86 (1.09-3.21) |  |
| 50– |  | 1.16 (0.87-1.53) |  |  | 0.85 (0.60-1.22) |  |  | 2.02 (1.25-3.27) |  |
| 60– |  | 1.00 (reference) |  |  | 1.00 (reference) |  |  | 1.00 (reference) |  |
| 70– |  | 0.95 (0.67-1.34) |  |  | 1.09 (0.71-1.66) |  |  | 0.61 (0.31-1.19) |  |
| ≥ 80 |  | 1.05 (0.51-2.13) |  |  | 1.17 (0.49-2.77) |  |  | 0.59 (0.12-2.28) |  |
| Artery of IA |  |  |  |  |  |  |  |  |  |
| ICA |  | 1.00 (reference) |  |  | 1.00 (reference) |  |  | 1.00 (reference) |  |
| VA |  | 1.14 (0.61-2.10) |  |  | 1.44 (0.63-3.39) |  |  | 0.96 (0.34-2.55) |  |
| BA |  | 0.85 (0.36-1.97) |  |  | 0.91 (0.28-2.90) |  |  | 0.92 (0.23-3.37) |  |
| ACoA |  | 1.70 (1.27-2.28) |  |  | 1.48 (1.00-2.19) |  |  | 2.12 (1.33-3.39) |  |
| ACA |  | 1.00 (0.63-1.58) |  |  | 0.82 (0.46-1.44) |  |  | 1.56 (0.70-3.51) |  |
| MCA |  | 0.95 (0.70-1.29) |  |  | 0.83 (0.56-1.21) |  |  | 1.26 (0.74-2.13) |  |
| PCA |  | 0.94 (0.36-2.42) |  |  | 0.72 (0.24-2.12) |  |  | 1.78 (0.26-15.37) |  |
| Other arteries |  | 1.01 (0.47-2.18) |  |  | 1.30 (0.54-3.30) |  |  | 0.45 (0.05-2.41) |  |
| Size, mm |  |  | 0.974 |  |  | 0.981 |  |  | 0.897 |
| ≤ 3 |  | 1.08 (0.42-2.81) |  |  | 0.71 (0.21-2.27) |  |  | 1.88 (0.33-14.88) |  |
| 3– |  | 1.00 (reference) |  |  | 1.00 (reference) |  |  | 1.00 (reference) |  |
| 5– |  | 1.52 (0.60-3.93) |  |  | 0.98 (0.30-3.10) |  |  | 2.97 (0.52-23.50) |  |
| 7– |  | 1.57 (0.61-4.09) |  |  | 0.98 (0.29-3.12) |  |  | 2.90 (0.50-23.24) |  |
| 9– |  | 1.63 (0.62-4.34) |  |  | 1.15 (0.33-3.75) |  |  | 2.86 (0.48-23.58) |  |
| 11– |  | 1.54 (0.56-4.29) |  |  | 1.17 (0.32-4.12) |  |  | 2.11 (0.33-18.18) |  |
| ≥ 13 |  | 1.11 (0.41-3.03) |  |  | 0.74 (0.21-2.54) |  |  | 1.91 (0.31-15.84) |  |
| Multiple IA |  |  | < 0.001 |  |  | < 0.001 |  |  | < 0.001 |
| Single |  | 1.00 (reference) |  |  | 1.00 (reference) |  |  | 1.00 (reference) |  |
| Multiple |  | 0.53 (0.40-0.69) |  |  | 0.55 (0.40-0.76) |  |  | 0.40 (0.23-0.70) |  |
| ICH history |  | 1.67 (1.26-2.22) |  |  | 1.87 (1.31-2.70) |  |  | 1.46 (0.89-2.39) |  |
| Ischemic stroke |  | 0.37 (0.28-0.48) |  |  | 0.39 (0.27-0.56) |  |  | 0.31 (0.19-0.50) |  |
| Hypertension |  | 2.45 (1.93-3.12) |  |  | 2.00 (1.48-2.71) |  |  | 3.85 (2.56-5.87) |  |

OR, odds ratio. CI, confidence interval. BA, Basilar artery. VA, Vertebral artery. ACoA, Anterior Communicating Artery. PCA, Posterior Cerebral Artery. MCA, Middle Cerebral Artery. ACA, Anterior Cerebral Artery. ICA, Internal Carotid Artery.

**NOTE.** Apart from sex, other variables are matched.

## **Supplementary Table 6.** Characteristics of intracranial aneurysms from patients with multiple and with single aneurysms by sex

| Characteristic |  | Overall |  |  |  | Multiple IA |  |  |  | Single IA |  |
| --- | --- | --- | --- | --- | --- | --- | --- | --- | --- | --- | --- |
|  | **Single IA**  **(n = 1463)** | **Multiple IA**  **(n = 960)** | ***P* value** |  | **Female**  **(n = 658)** | **Male**  **(n =302)** | ***P* value** |  | **Female**  **(n = 831)** | **Male**  **(n = 632)** | ***P* value** |
| Sex, n (%) |  |  | <0.001 |  |  |  |  |  |  |  |  |
| Female | 831 (56.80) | 658 (68.54) |  |  |  |  |  |  |  |  |  |
| Male | 632 (43.20) | 302 (31.46) |  |  |  |  |  |  |  |  |  |
| Age, median[IQR], years | 61.00 [52.00, 69.00] | 65.00 [56.00, 73.00] | <0.001 |  | 65.00 [57.00, 73.00] | 64.00 [55.00, 72.00] | 0.091 |  | 62.00 [53.00, 69.00] | 60.00 [51.00, 68.00] | 0.271 |
| Age, n (%), years |  |  | < 0.001 |  |  |  | 0.147 |  |  |  | 0.377 |
| ≤ 40 | 58 (3.99) | 23 (2.40) |  |  | 16 (2.44) | 7 (2.32) |  |  | 29 (3.52) | 29 (4.61) |  |
| 40– | 252 (17.33) | 92 (9.60) |  |  | 54 (8.23) | 38 (12.58) |  |  | 137 (16.61) | 115 (18.28) |  |
| 50– | 396 (27.24) | 227 (23.70) |  |  | 147 (22.41) | 80 (26.49) |  |  | 223 (27.03) | 173 (27.50) |  |
| 60– | 444 (30.54) | 318 (33.19) |  |  | 230 (35.06) | 88 (29.14) |  |  | 255 (30.91) | 189 (30.05) |  |
| 70– | 239 (16.44) | 252 (26.30) |  |  | 176 (26.83) | 76 (25.17) |  |  | 148 (17.94) | 91 (14.47) |  |
| ≥ 80 | 65 (4.47) | 46 (4.80) |  |  | 33 (5.03) | 13 (4.30) |  |  | 33 (4.00) | 32 (5.09) |  |
| Artery of IA, n (%) |  |  | < 0.001 |  |  |  | 0.854 |  |  |  | < 0.001 |
| ICA | 672 (45.93) | 554 (57.71) |  |  | 386 (58.66) | 168 (55.63) |  |  | 432 (51.99) | 240 (37.97) |  |
| VA | 47 (3.21) | 27 (2.81) |  |  | 18 (2.74) | 9 (2.98) |  |  | 22 (2.65) | 25 (3.96) |  |
| BA | 38 (2.60) | 40 (4.17) |  |  | 23 (3.50) | 17 (5.63) |  |  | 17 (2.05) | 21 (3.32) |  |
| ACoA | 294 (20.10) | 79 (8.23) |  |  | 53 (8.05) | 26 (8.61) |  |  | 126 (15.16) | 168 (26.58) |  |
| ACA | 115 (7.86) | 34 (3.54) |  |  | 24 (3.65) | 10 (3.31) |  |  | 68 (8.18) | 47 (7.44) |  |
| MCA | 236 (16.13) | 160 (16.67) |  |  | 108 (16.41) | 52 (17.22) |  |  | 131 (15.76) | 105 (16.61) |  |
| PCA | 29 (1.98) | 37 (3.85) |  |  | 27 (4.10) | 10 (3.31) |  |  | 16 (1.93) | 13 (2.06) |  |
| Other arteries | 32 (2.19) | 29 (3.02) |  |  | 19 (2.89) | 10 (3.31) |  |  | 19 (2.29) | 13 (2.06) |  |
| Size, median[IQR], mm | 6.80 [4.90, 9.55] | 5.70 [4.10, 8.53] | <0.001 |  | 5.80 [4.20, 8.78] | 5.50 [4.03, 8.07] | 0.378 |  | 6.90 [5.05, 9.60] | 6.60 [4.80, 9.50] | 0.231 |
| Size, n (%), mm |  |  | <0.001 |  |  |  | 0.741 |  |  |  | 0.297 |
| ≤ 3 | 38 (2.60) | 76 (7.92) |  |  | 55 (8.36) | 21 (6.95) |  |  | 20 (2.41) | 18 (2.85) |  |
| 3– | 351 (23.99) | 313 (32.60) |  |  | 203 (30.85) | 110 (36.42) |  |  | 188 (22.62) | 163 (25.79) |  |
| 5– | 386 (26.38) | 223 (23.23) |  |  | 156 (23.71) | 67 (22.19) |  |  | 219 (26.35) | 167 (26.42) |  |
| 7– | 271 (18.52) | 132 (13.75) |  |  | 91 (13.83) | 41 (13.58) |  |  | 160 (19.25) | 111 (17.56) |  |
| 9– | 156 (10.66) | 92 (9.58) |  |  | 65 (9.88) | 27 (8.94) |  |  | 101 (12.15) | 55 (8.70) |  |
| 11– | 90 (6.15) | 41 (4.27) |  |  | 29 (4.41) | 12 (3.97) |  |  | 52 (6.26) | 38 (6.01) |  |
| ≥ 13 | 171 (11.69) | 83 (8.65) |  |  | 59 (8.97) | 24 (7.95) |  |  | 91 (10.95) | 80 (12.66) |  |
| Rupture, n (%) | 587 (40.12) | 147 (15.31) | < 0.001 |  | 119 (18.09) | 28 (9.27) | < 0.001 |  | 356 (42.84) | 231 (36.55) | 0.017 |
| Hypertension, n (%) | 583 (39.85) | 417 (43.44) | 0.087 |  | 290 (44.07) | 127 (42.05) | 0.606 |  | 324 (38.99) | 259 (40.98) | 0.473 |
| ICH history, n (%) | 147 (8.70) | 170 (23.16) | 0.893 |  | 87 (13.22) | 37 (12.25) | 0.755 |  | 113 (13.60) | 80 (12.66) | 0.654 |
| Ischemic stroke, n (%) | 515 (30.55) | 134 (18.26) | <0.001 |  | 195 (29.77) | 100 (33.11) | 0.335 |  | 196 (23.59) | 158 (25.00) | 0.573 |


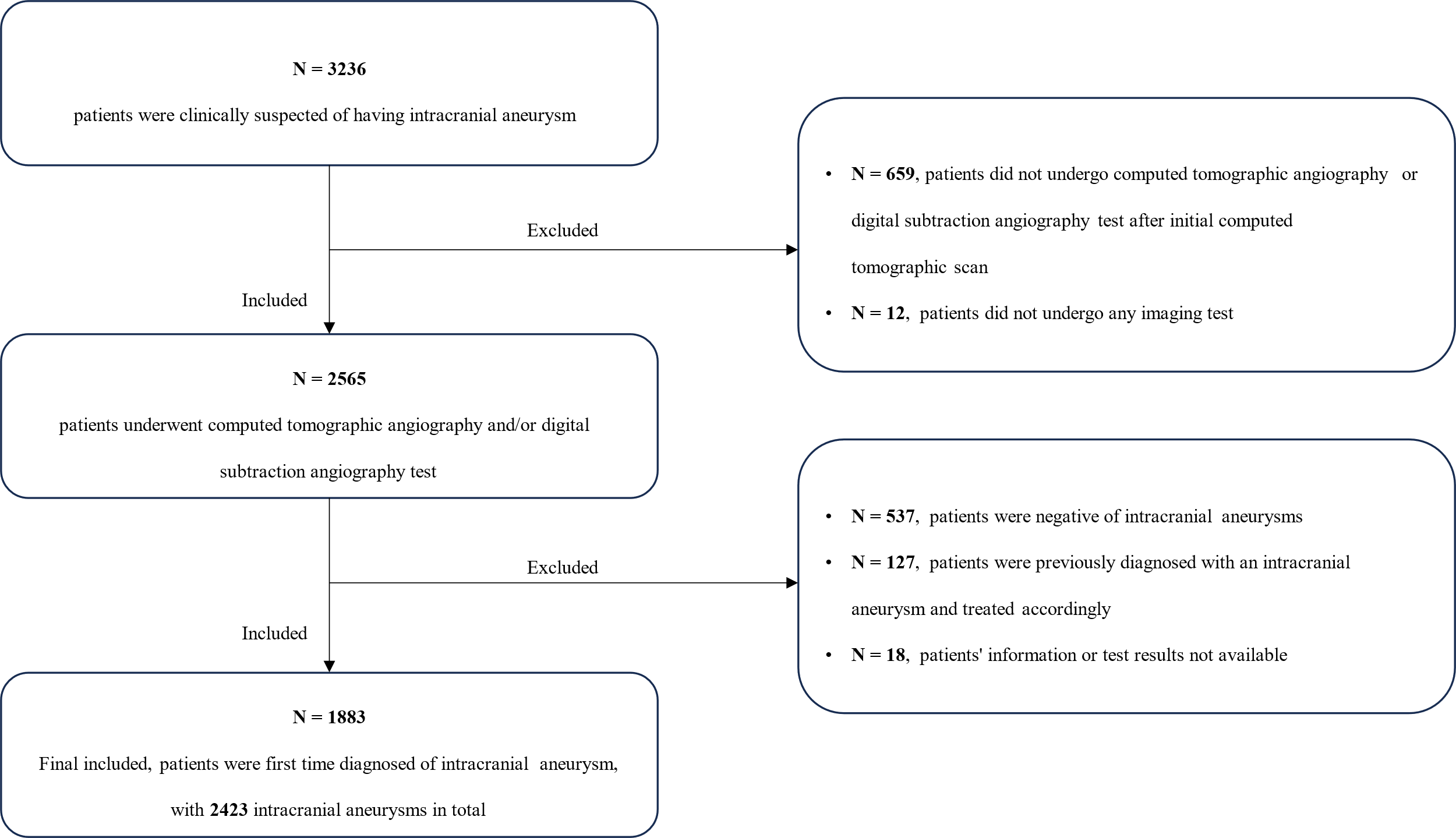


## Supplementary Figure 1. Flowchart of patient inclusion

This chart shows how the patient inclusion process was conducted; 1883 patients with 2423 intracranial aneurysm were included.

## Supplementary Figure 2. Non-linear association between size of intracranial aneurysms and rupture by age in different sex

Legend: This plot shows size change associated with intracranial aneurysms (IA) rupture across different artery by female and male, an n-shape association was found in female 50-60 group, 60-70group ,70-80group and in male 70-80group, ICA. Odds ratio(OR) and 95%confidence interval (95%CI) is calculated based on per unit(mm) change using restrict cubic spline and adjusted for history of intracerebral hemorrhage, history of ischemic stroke, hypertension, IA multiplicity and patient age. Peak OR is the highest OR as size changed. Density of IA counts is displayed on log10 scale. (T1) 40-50 years group of total IAs, (T2) 50-60 years group of total IAs , (T3) 60-70 years group of total IAs , (T4) 70-80 years group of total IAs,(F1) 40-50 years group of female IAs , (F2) 50-60 years group of female IAs , (F3) 60-70 years group of female IAs , (F4) 70-80 years group of female IAs,(M1) 40-50 years group of male IAs, (M2) 50-60 years group of male IAs , (M3) 60-70 years group of male IAs , (M4) 70-80 years group of male IAs.


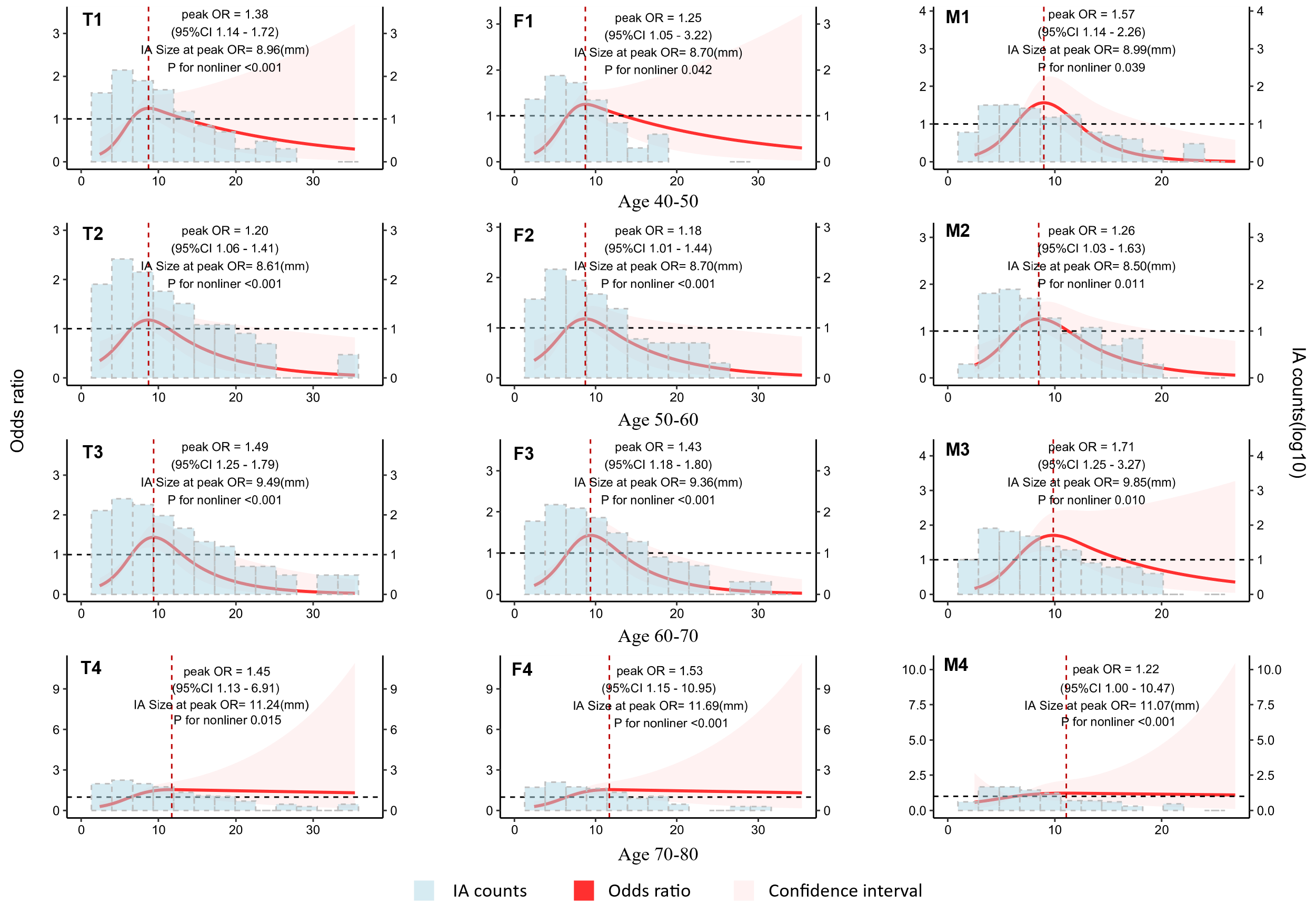


## Supplementary Figure 3. Pairwise comparisons of intracranial aneurysm size across each artery

Legend: This plot shows result of pairwise comparison of IA in each artery. Comparison is based on independent T test, *P* value was adjusted by Bonferroni method.


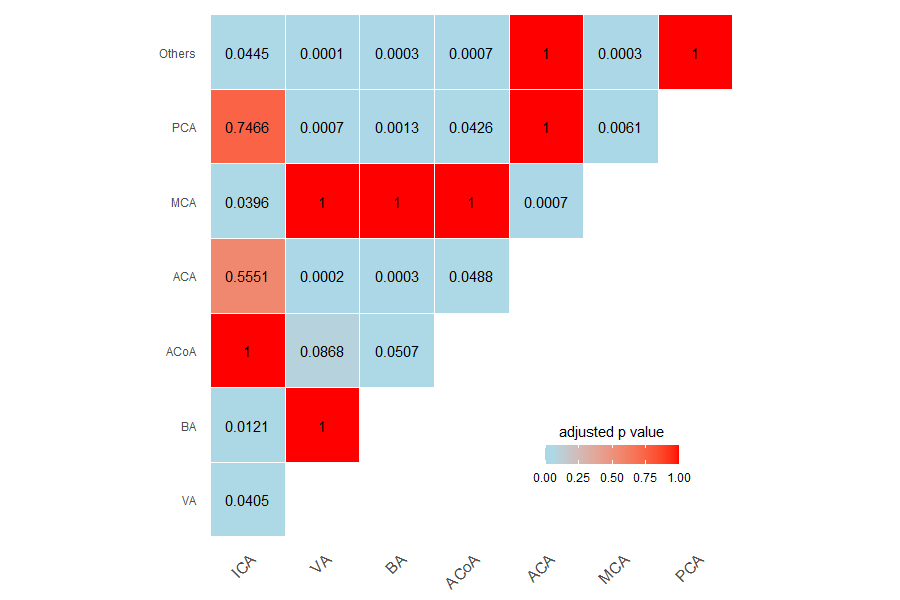


## Supplementary Figure 4. E-value plot of association between female sex and intracranial aneurysm rupture

Legend: This plot shows minimum E-value as sensitivity analysis on unadjusted confounder that may reverse the main founding, that a unadjusted confounder with an Odds ratio larger than 2.83 may reverse current founding. Each point along the curve defines a joint relationship between the two sensitivity parameters that could potentially explain away the estimated effect. If one of the two parameters is smaller than the E-value, the other must be larger, as defined by the plotted curve. This analysis used <https://www.evalue-calculator.com/evalue/>


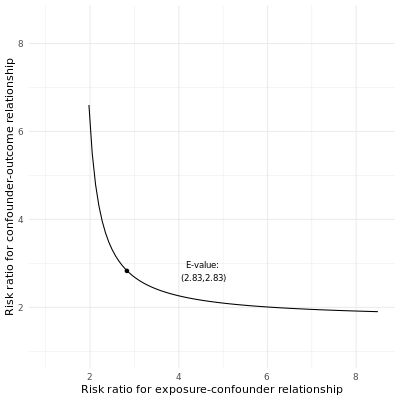


## Supplementary Figure 5. Change in effect estimate of female sex of different confounding variables

Legend: This plot shows the changes when potential confounding factors are sequentially added to the model in a stepwise fashion. At each step, one variable which creates the largest change (%) of the effect estimate among the remaining variables is added to the model. Crude refers to the basic model “IA rupture ~ female sex”.


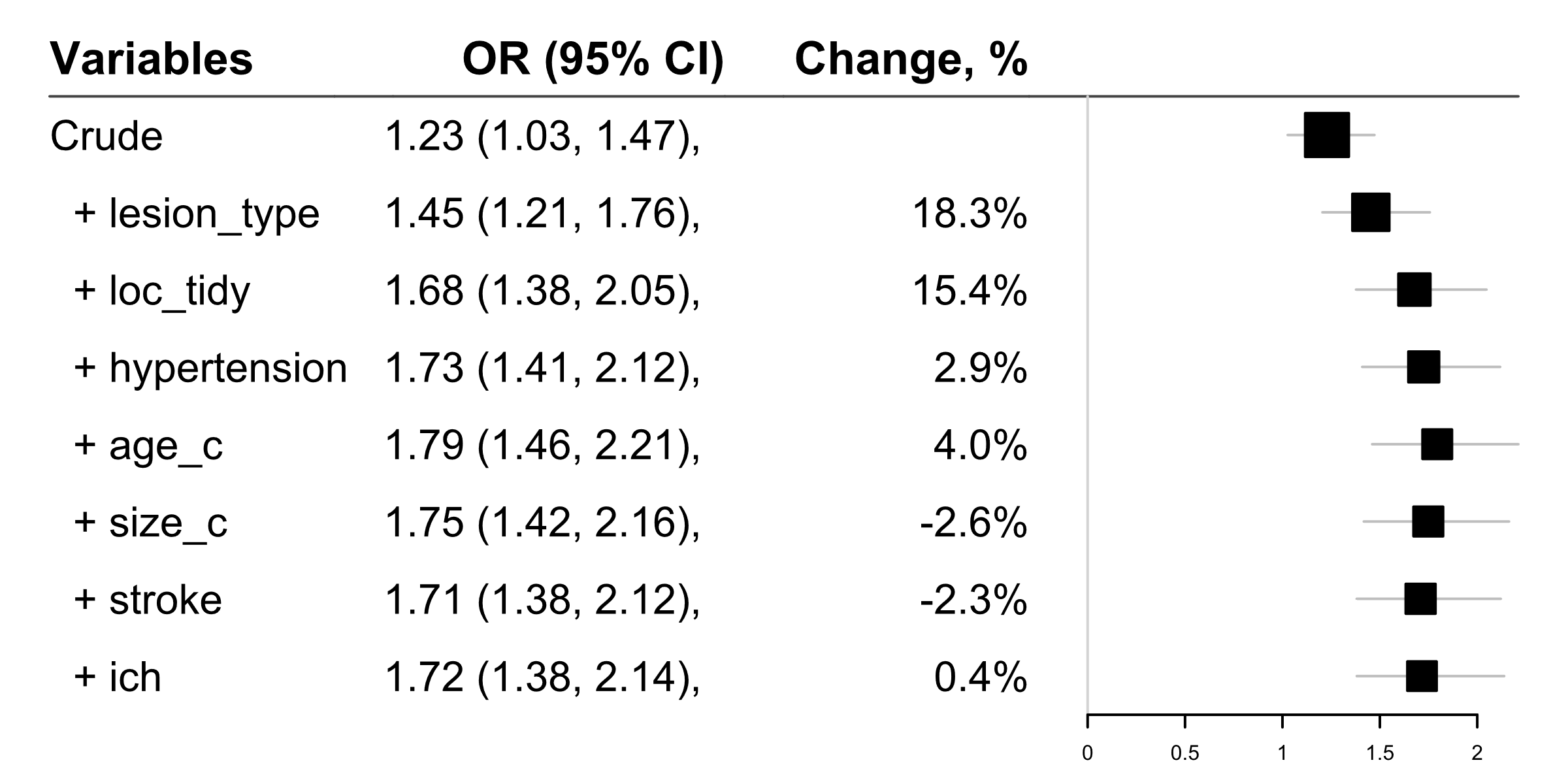

Supplement: Supplementary file 1 [file Table_1.DOCX]
